# Virtual Reality for Outreach and Community‐Engagement in Paleogenomics: Development of a 360° Tour of an Ancient DNA Laboratory

**DOI:** 10.1002/ece3.74033

**Published:** 2026-07-17

**Authors:** Christina M. Balentine, Alisha Khalil, Abigail Sink, Horvey Palacios, Deborah A. Bolnick, Michael Lavin, Dave Givens, Chuck Durfor, Raquel E. Fleskes

**Affiliations:** ^1^ Department of Anthropology Dartmouth College Hanover New Hampshire USA; ^2^ Department of Anthropology University of Connecticut Storrs Connecticut USA; ^3^ Department of Anthropology University of Oklahoma Norman Oklahoma USA; ^4^ Institute for Systems Genomics University of Connecticut Storrs Connecticut USA; ^5^ Jamestown Rediscovery Foundation Williamsburg Virginia USA; ^6^ TerraSearch Geophysical Williamsburg Virginia USA

**Keywords:** ancient DNA, community engagement, digital visualization, paleogenomics, science communication, virtual reality

## Abstract

Virtual reality (VR) and digital visualization tools offer new opportunities for science education and community outreach by increasing the accessibility and transparency of controlled‐access laboratory spaces, such as ancient DNA (aDNA) cleanroom environments for paleogenomics research. These visualization methods provide a critical intervention for the field of paleogenomics by combining public science outreach efforts with digital tools to support engagement with descendant communities, the public, or other communities of care. Here, we present the creation of a 360° Tour of the Ancient DNA Laboratory at the University of Connecticut (360° Tour). The virtual environment was developed using 3DVista software and hosts interactive audio, images, and videos that allow users to engage with aDNA laboratory workflows in the spaces where they are executed. Interactive elements are organized into three thematic categories: Orientation Audio, which introduces the laboratory spaces and their functions; Information Hotspots, which provide contextual information about equipment or processes; and Embedded Skills Videos, which demonstrate core aDNA methods. We present the 360° Tour's web integration and detail its use for public outreach in collaboration with Jamestown Rediscovery Foundation's Bioarchaeology Program. Overall, VR tools such as the 360° Tour offer a transferable framework to improve accessibility and transparency in laboratory research, enhancing community engagement and education efforts for paleogenomics and science more broadly.

## Graphical abstract

Virtual reality and digital visualization tools offer new opportunities for science education and community outreach by increasing the accessibility and transparency of controlled‐access laboratory spaces. Here, we present the creation of a 360° Tour of the Ancient DNA Laboratory at the University of Connecticut, created in collaboration with the Jamestown Rediscovery Foundation's Bioarchaeology Program. We evaluate how virtual technologies, such as those utilized in the tour, can advance accessible and transparent science, and why these principles are foundational to developing ethical scientific practices.

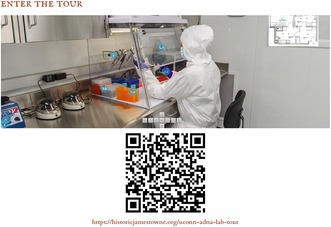

## 1 Introduction

Contemporary research practices increasingly emphasize the importance of public engagement, yet many aspects of how scientific knowledge is produced remain inaccessible to nonspecialist audiences (Arango‐Isaza et al. 2023; Brockie et al. 2022; de la Cova et al. 2024; Fleskes et al. 2022; Kowal et al. 2023; Stilgoe et al. 2014; Wagner et al. 2020). This is especially true for laboratory‐based research conducted in controlled‐access environments, where physical restrictions hinder visibility and complex workflows can be difficult to clearly explain (Arango‐Isaza et al. 2023; Fleskes et al. 2025). However, effectively communicating research methods and explaining important features of laboratory research environments are essential in public science outreach that aims to foster understanding of how scientific results are produced (Besley and Dudo 2022; National Academies of Sciences, Engineering, and Medicine 2017). Increasing the accessibility of research processes is therefore critical not only for effectively communicating scientific findings but also to facilitate broader understanding of how scientific knowledge is produced.

These challenges are especially pronounced in paleogenomics, which analyzes ancient DNA (aDNA) to investigate past population histories and evolutionary processes of adaptation (Ávila‐Arcos et al. 2023; Kaestle and Horsburgh 2002; Marciniak and Perry 2017; Orlando et al. 2021). Paleogenomic research requires positive‐pressured cleanroom laboratory facilities to carry out complex, multiday workflows designed to maximize recovery of highly degraded DNA while minimizing contamination from outside sources (Fulton and Shapiro 2019; Llamas et al. 2017). These workflows involve numerous steps, from sample preparation, DNA extraction, DNA library preparation, sequencing, and bioinformatic analysis (Kaestle and Horsburgh 2002; Llamas et al. 2017), making the research process difficult to clearly convey to nonspecialist audiences (Fleskes et al. 2025). At the same time, paleogenomic research involving human ancestral remains (“Ancestors”) often carries ethical, cultural, or spiritual significance for descendant communities and other connected communities of care (Fleskes et al. 2022; Halmai et al. 2025; Kowal et al. 2023; Wagner et al. 2020). In these contexts, limited visibility around research methods and laboratory facilities can complicate efforts to effectively communicate research practices and expectations in order to build trust and understanding, underscoring the need for tools that support greater transparency and accessibility about the research process.

Addressing these barriers requires tools that can make restricted laboratory spaces and complex research workflows visible, understandable, and engaging to nonspecialist audiences. Virtual reality (VR) technology provides one such approach. VR immerses users in a three‐dimensional interactive environment, allowing users to look, move, and interact as though they were physically present within a real‐world space (Calil et al. 2021; Mulders et al. 2020). The immersive properties of VR have been adapted for use in classrooms (Archibald et al. 2025; Carmona‐Galindo et al. 2025; Geng and Su 2024; Gestiada et al. 2025) and other learning environments, including museums (Bachiller et al. 2023; Banfi et al. 2023), cultural heritage sites (Bruno et al. 2018; Wessels et al. 2014), laboratories (Levonis et al. 2021; McCaslin et al. 2014), and public outreach events (Nelson et al. 2020). A growing body of literature has shown that VR technology in the classroom can increase engagement and comprehension (Archibald et al. 2025; Carmona‐Galindo et al. 2025). Notably, VR can facilitate interactions with otherwise inaccessible or difficult‐to‐access environments, such as heritage sites like Petra, Jordan (Wessels et al. 2014), coral reefs impacted by climate change (Nelson et al. 2020), the effects of sea‐level rise for coastal communities (Calil et al. 2021), or other places where laboratory research takes place (Levonis et al. 2021).

Virtual technologies also play an important role in creating opportunities to introduce niche scientific fields to individuals and communities who have historically been excluded due to geographic, institutional, educational, or structural barriers (Gallardo‐Williams and Dunnagan 2024). Demographic data from the National Science Foundation indicate that women, African American, and Hispanic populations remain persistently underrepresented in STEM fields (National Science Board 2024, 16–18). In 2021, the ratio of men to women in STEM‐related professions was approximately 8.5 to 1, and African American and Hispanic representation in STEM professions was three percentage points lower than their representation in the general workforce (National Science Board 2024, 7). Virtual technologies can be used to help address these disparities by enabling scientists to reach underrepresented groups through broader dissemination of scientific methods and findings (Swartz et al. 2019).

**FIGURE 1:**
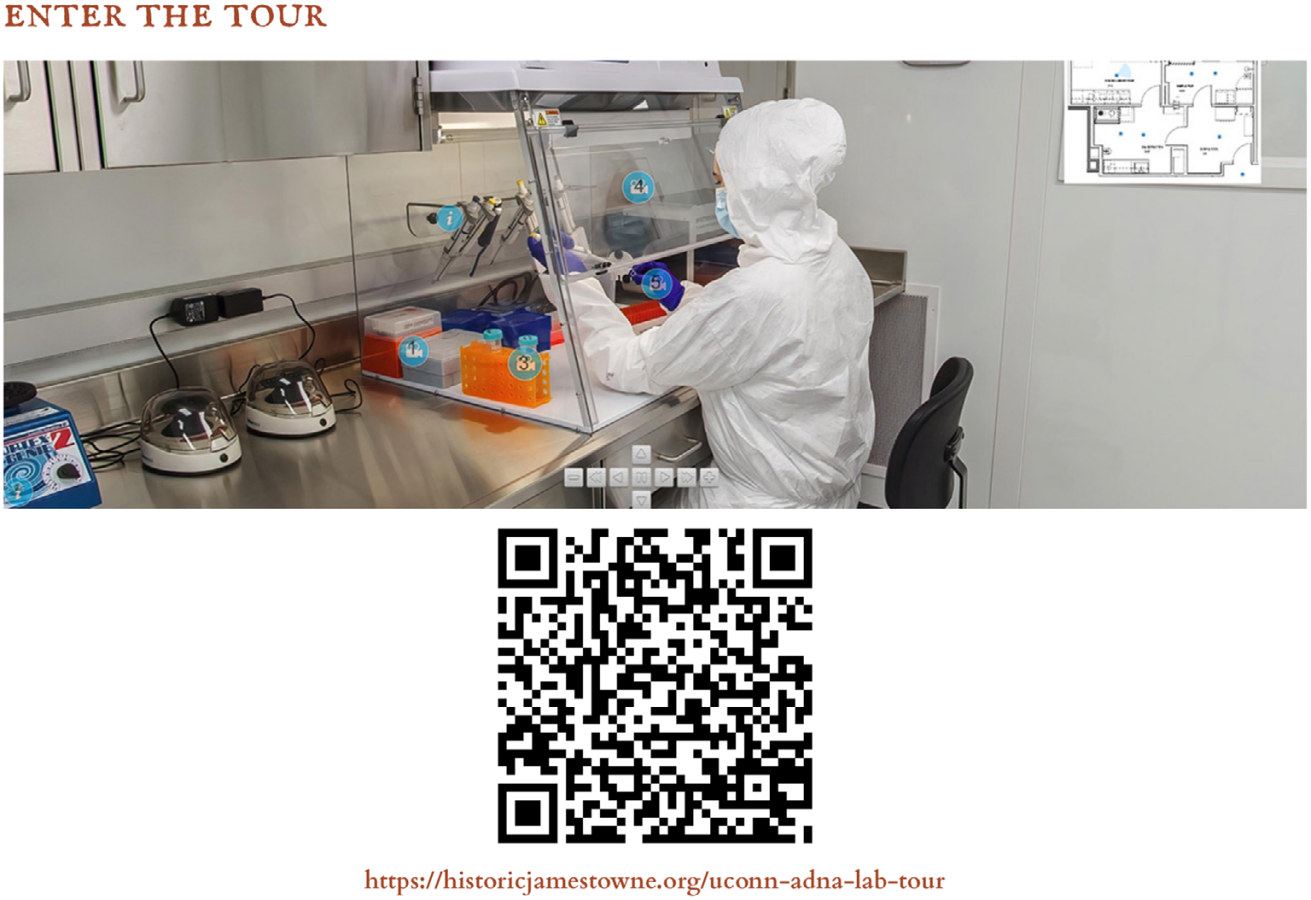
QR code for directly accessing the 360° Tour on the Jamestown Rediscovery Foundation's Historic Jamestowne website.

Together, the applications described above suggest that VR may offer a useful strategy to increase public engagement with paleogenomics research. Here, we present the development and implementation of a 360° Virtual Reality Tour of the University of Connecticut Ancient DNA Laboratory (360° Tour) in collaboration with the Jamestown Rediscovery Foundation. The 360° Tour was developed for public outreach associated with the 1607 Burial Ground Project, which uses aDNA to reconstruct the lived histories of the original English colonists involved in Jamestown's initial settlement. The 360° Tour is available online^1^ and can be accessed by scanning the QR code in Figure 1.

In this manuscript, we describe the development, components, and implementation of the 360° Tour, and evaluate its role as a tool for increasing accessibility to controlled laboratory environments and transparency in paleogenomic research practices. We further discuss how such approaches can support ethical engagement with descendant communities and the broader public in paleogenomics, particularly with respect to informed consent, data governance, and equity. Overall, this work demonstrates how VR technologies can be leveraged to increase accessibility and transparency in the research process.

## 2 Creation of the 360° Tour

In what follows, we describe the tools used and steps taken in creating the 360° Tour. We first detail the selection of samples for workflow demonstrations and provide a written overview of the cleanroom environment within the Ancient DNA Laboratory at the University of Connecticut (UConn). Next, we provide an overview of the specific tools and programs used to construct the 360° Tour. Components of the tour include panorama photographs, audio, video, and images, which were assembled into the tour using 3Dvista Virtual Tour Pro software. The recording of the tour was completed in 2 days, and the process of assembling the tour took 4 weeks using a MacBook Pro M2 Max computer (Durfor 2024). The total cost of this effort included the cost of the cameras and microphones, as all other computer software was free, available for a free trial, or available at a discount with a university subscription.

### 2.1 Proxy Samples Used for Workflow Demonstrations

To avoid unnecessary handling or testing of archaeological human Ancestors and to respect the cultural sensitivity of human remains, bovine bone or sterile water samples were used for all demonstrations within the 360° Tour. These substitutes enabled the visualization of key stages of the aDNA workflow to meet the project's educational objectives without involving human skeletal remains. All demonstrations were conducted following standard aDNA laboratory protocols and contamination prevention procedures to maintain authenticity of the workflow presented, as outlined below.

### 2.2 Contamination Controls

The Ancient DNA Laboratory at UConn is a restricted‐access ISO 7/6 cleanroom facility with stringent contamination control protocols in place to prevent the introduction of exogenous DNA, or DNA from outside sources (Llamas et al. 2017). Both the researcher (Fleskes) and videographer (Durfor) wore full Tyvek suits, face masks, hair nets, and two sets of gloves throughout filming. The camera tripod was decontaminated first with *DNA Away* (Thermo Scientific), followed by 70% ethanol applied using Kimwipes (Kimberly‐Clark Professional). During filming, the videographer stayed in the center of each room and avoided contact with laboratory surfaces unless necessary. The only surface affected was the floor where the bottom of the camera tripod was positioned. Following filming, all laboratory surfaces were fully decontaminated using *DNA Away* and 70% ethanol, and overhead UV lights (254 nm) were activated overnight.

### 2.3 Photo Collection of 360° Photography Panoramas

At each location, a full‐frame Pentax K‐1 camera and an 8 mm lens mounted on a tripod captured four photographs at 90‐degree intervals. The camera was secured on an L‐Bracket attached to a 140 mm Rail Slide Metal Quick Release Clamp, mounted atop a metal 2.4 in. (60 mm) quick‐release plate and a panoramic panning base with Arca Swiss style plate. This configuration ensured that the four images were level and equally spaced for panoramic stitching.

### 2.4 Post‐Processing and 360° Equirectangular Panorama Assembly

Image clarity was enhanced using Topaz Denoise software, and the visible portions of the tripod were removed using the clone stamp tool in Adobe Photoshop. The resulting TIFF images were stitched into a single 360° equirectangular panorama using PTGui software. Final panoramas (approximately 60–70 MB each) were exported and saved in JPG format.

### 2.5 Audio Recordings

Orientation Audio for introducing each room of the 360° Tour was recorded to provide narrated introductions and contextual explanations of laboratory spaces and their functions (Table 1). Audio recordings were produced using iMovie (version 10.4.3) and captured in a quiet indoor setting to ensure sound clarity. The recordings were edited for volume normalization and background noise reduction prior to integration into the VR tour. Final audio files were embedded within the tour and programmed to play automatically upon entry into each corresponding room.

**TABLE 1:** Audio transcripts of the Orientation Audio voiceovers heard when entering each room in the 360° Tour.

Area	Orientation transcript
Exterior of ancient DNA Lab	“Welcome to the Ancient DNA Lab at the University of Connecticut. This is the outside of our lab space, where we prepared to enter into the inner gowning room.”
Gowning room	“This is the gowning room of the Ancient DNA Lab. This room serves as the halfway point, where we put on personal protective equipment, such as gloves and Tyvek suits, in order to enter into the inner suites.”
Inner chamber 1 (Sample preparation lab)	“You have entered into the first inner chamber of the Ancient DNA Lab. This is the sample preparation suite. In this suite we prepare bone or teeth samples for DNA extraction.”
Inner chamber 2 (Extraction lab)	“You are now in our second inner chamber of the Ancient DNA Lab. This is the DNA extraction suite. In this chamber, DNA extractions are conducted.”
Inner chamber 3 (Library & PCR preparation lab)	“This is the third and final inner chamber of the Ancient DNA Lab. This is the library preparation suite. In this chamber, we take our extracted DNAs and prepare them for sequencing.”
Sequencing center	“This the University of Connecticut's Center for Genome Innovation. Here at this sequencing center, our DNA libraries are read by a sequencing machine in order to obtain DNA sequence information.”

### 2.6 Information Hotspot Selection

Informational Hotspots were selected to highlight laboratory equipment, materials, and procedures that are central to aDNA workflows (Table 2). Priority was given to tools and spaces involved in contamination control, sample preparation, DNA extraction, and library preparation, as these represent critical stages of the paleogenomic research process. Hotspots were designed to provide brief visual and textual explanations that contextualize each item's role within the broader laboratory workflow. This approach ensured that the tour emphasized scientifically relevant features of the laboratory while supporting user understanding of how individual components contribute to the generation of aDNA data.

**TABLE 2:** List of information hotspots in the 360° Tour.

Area	Information hotspots	Accompanying descriptive text
Exterior of Ancient DNA Lab	1: Shoe Rack and Black Crocs	“To reduce possible contamination, street shoes are removed and Black Lab Crocs are worn”
	2: A Pack of Sterile Gloves	“Before you enter the lab, you put on a pair of sterile gloves”
	3: A Bottle of *DNA Away*	“Once Black Lab Crocs and gloves are put on, contaminants on the gloves are removed by DNA AWAY, (a dilute alkaline solution) that removes DNA from equipment that would be damaged by a 10% bleach solution”
	4: Entry Checklist	—
Gowning Room	1: Shoe Rack and White Crocs	“The Black Lab Crocs are removed during gowning and replaced by White Lab Crocs to enter the inner chambers”
	2: UV Lights	“These lights prevent any modern DNA from being amplified to help prevent contamination”
	3: Sterile Gloves	“While gloves are put on before entering the gowning room, a second set of gloves are put on as the final step in gowning”
	4: 10% Bleach	“A 10% Bleach Solution is frequently sprayed on gloves to remove DNA contamination”
	5: 70% Ethanol	“A 70% Ethanol Solution is sprayed on gloves to remove the 10% Bleach Solution”
	6: *DNA Away*	“DNA Away (a dilute alkaline solution) removes DNA contamination from tools that cannot withstand bleach.”
	7–8: Inner Chamber 1 Doorplate & Checklist	—
	9–10: Inner Chamber 2 Doorplate & Checklist	—
	11: Laboratory Safety Card	—
	12: BSL1 Card	—
	13: Storage Drawers for Masks, Hairnets	—
	14: Storage Drawers for Pipettes and Pipette Tips	—
	15: Storage Drawers for Misc. Lab Supplies	—
	16: Storage Drawers for Kimwipes, Spray Bottles	—
	17: Checklist for Leaving the Lab	—
Inner Chamber 1 (Sample Preparation Lab)	1: Laminar Flow Hood	“A laminar flow hood protects against dust and other potential contaminants via a constant, unidirectional flow of air inside the hood. It also reduces the worker's exposure to harmful materials used inside the hood”
	2: Microbalance	“The weight of bone powder samples and reagents is measured with a Microbalance”
	3: Bone Dust Tools	“These tools are used to turn pieces of bone into bone dust”
	4: Hazardous Waste Container	“Careful disposal of hazardous waste is critical for lab safety and high quality study results”
	5: Cleaning Instructions	—
	6–7: Eyewash/Shower Station & Sign	“In case of an accident, the eyewash provides a rapid method of flushing harmful chemicals from the eyes”
	8: Checklist for Leaving Inner Chamber 1	—
Inner Chamber 2 (Extraction Lab)	1: Pipettes	“Pipettes (above) with disposable tips (on the right) are used to rapidly and accurately measure microliter quantities of reagents for aDNA preparation”
	2: Large Centrifuge	“This large centrifuge spins samples rapidly to allow DNA to be separated in extraction”
	3: Flammable Storage Cabinet	“Acid and Flammable Storage Cabinets provide a safe way to store dangerous materials”
	4: Acid Storage Cabinet	“Acid and Flammable Storage Cabinets provide a safe way to store dangerous materials”
	5: Inner Chamber 3 Doorplate	“Library & PCR preparation laboratory”
	6: Heating Block	“The heating block provides a controlled heated environment for sample preparation”
	7: Checklist for Leaving Inner Chamber 2	—
	8: Sink	“Time to do the dishes with 10% Bleach and 70% ethanol. Lab dishes are soaked in the bucket in a 10% bleach solution for decontamination”
	9: Vortexer	“Vibrations created by pressing a sample vial on the top of a Vortex Genie rapidly mixes solutions”
	10: UV Crosslinker	“Within the UV Crosslinker, supplies and sample vials are decontaminated using high intensity UV light”
	11: Cabinet of Reagents	—
	12–13: Eyewash/Shower Station & Sign	“In case of an accident, the eyewash provides a rapid method of flushing harmful chemicals from the eyes”
Inner Chamber 3 (Library & PCR Preparation Lab)	1: Vortexer	“Vibrations created by pressing a sample vial on the top of a Vortex Genie rapidly mixes solutions”
	2: Pipettes	“Pipettes (above) with disposable tips (on the right) are used to rapidly and accurately measure microliter quantities of reagents for aDNA preparation”
	3: Checklist for Leaving Inner Chamber 3	—
	4: Centrifuge	“The centrifuge gently precipitates material from solution by spinning at a high speed”
	5: Freezer	“Temperature‐sensitive reagents and samples are stored in a −20°C freezer”
Sequencing Center	1: Illumina MiSeq Sequencing Machine	“This is an Illumina MiSeq Sequencing Machine, which images the bases (A, G, C, T) in our DNA to generate sequencing data”

Abbreviations: BSL1, biosafety level 1; UV, ultraviolet.

### 2.7 Embedded Skills Video Development

Embedded Skills Videos were developed to document and demonstrate key stages of the aDNA laboratory workflow, including sample preparation, DNA extraction, damage repair, and library preparation (Table 3). Both 360° and standard 4K videos were recorded using GoPro Hero9, GoPro Max, and Sony FDR‐AX100 4K cameras, respectively. Audio was recorded with a Sennheiser microphone and a Sennheiser wireless transmitter/receiver system. All videos and audio files were edited using Adobe Premiere Pro, Adobe Premiere Rush, or iMovie (version 10.4.3) software due to differing software availabilities at the time of creation. Video captions were generated using Turboscribe AI and integrated into the final videos added using Adobe Premiere Rush.

**TABLE 3:** List of Embedded Skills Videos in the 360° Tour.

Area	Embedded skills videos
Exterior of Ancient DNA Lab	1: aDNA Overview
Gowning Room	1: aDNA Research at Jamestown
	2: Ethical Design of aDNA Studies
	3: PPE Gowning
Inner Chamber 1 (Sample Preparation Lab)	1: Decontamination Using UV Crosslinker
	2: Preparing Bone Samples for aDNA Processing Part 1
	3: Preparing Bone Samples for aDNA Processing Part 2
Inner Chamber 2 (Extraction Lab)	1: Beginning DNA Extraction
	2: DNA Incubation
	3: Making the Binding Buffer
	4: Spinning Down Samples in Centrifuge
	5: DNA Isolation
Inner Chamber 3 (Library & PCR Preparation Lab)	1: Removing Ancient DNA Damage – UDG Treatment
	2: Removing Ancient DNA Damage – Incubation
	3: Removing Ancient DNA Damage – UGI Treatment
	4: End Repair and Adapter Ligation
	5: PCR Preparation
Sequencing Center	1: From aDNA Sequence to Beyond

Abbreviations: PCR, polymerase chain reaction; PPE, personal protective equipment; UDG, Uracil‐DNA glycosylase; UGI, Uracil glycosylase inhibitor.

### 2.8 VR Tour Assembly

The VR Tour was assembled using 3DVista Virtual Tour Pro software. First, 360° equirectangular panoramas were uploaded into a 3DVista project and linked using the ‘hotspot’ function to establish the skeleton of the tour. The same function was then used to embed additional content, including videos, photographs, and audio recordings. Video files were uploaded to YouTube and linked in the VR tour via URL hotspots. A two‐dimensional floor plan representing the spatial layout of the laboratory was subsequently added, allowing users to orient their location in the tour and navigate between locations. The completed VR tour was exported in Mac and PC formats for offline use, and a web‐based version was uploaded to the Jamestown Rediscovery Foundation's Historic Jamestowne website^1^ (Video 1).

**VIDEO 1:** Walkthrough of the 360° Tour. Video content can be viewed at https://onlinelibrary.wiley.com/doi/10.1002/ece3.74033.

### 2.9 Accessibility

Given that the 360° Tour is primarily visual and may not be fully accessible to persons who are blind or have low vision, we intentionally utilized overhead audio and created subtitles for the Embedded Skills Videos. In addition, a textual description of the tour is provided in the supplemental materials of this manuscript (Appendix S1), which will be linked on the tour's webpage. These measures are intended to improve accessibility and ensure that key informational content is available through multiple formats.

## 3 Description of the 360° Tour

**FIGURE 2:**
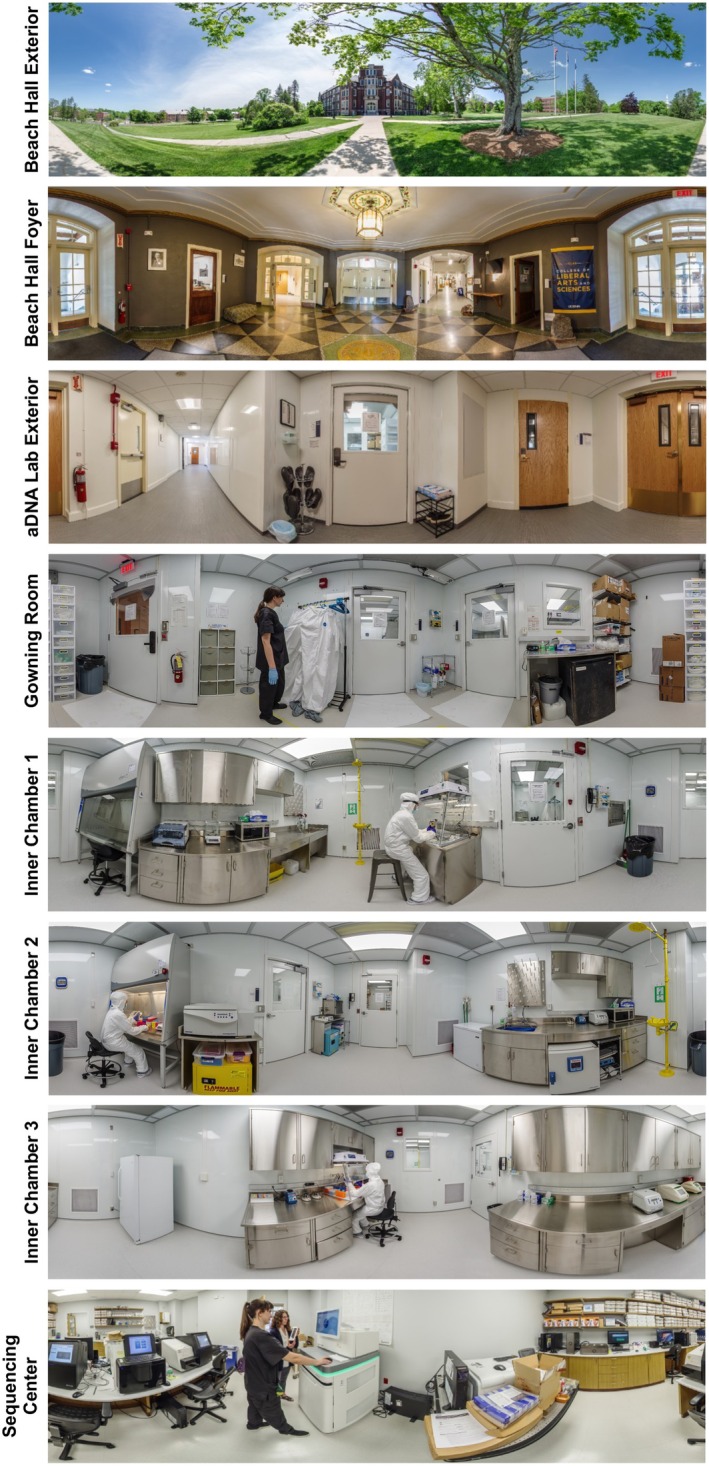
360° photographs comprising the eight views of the 360° Tour.

The 360° Tour consists of eight panoramic views that allow users to navigate the Ancient DNA Laboratory as if physically present in the space (Figure 2, Video 1). The tour begins outside Beach Hall on the UConn's campus, where the Ancient DNA Laboratory is located. Users then enter the building to view the first floor foyer before proceeding upstairs to the laboratory entrance. From here, users can enter the laboratory and move between four laboratory rooms, the Gowning Room and Inner Chambers 1, 2, and 3, which correspond to where various stages of the aDNA testing process take place. The tour concludes at the UConn's Center for Genome Innovation sequencing center, where users learn about aDNA sequencing and the downstream computational analyses.

The eight views were selected to illustrate the spatial organization and workflow of a paleogenomics laboratory. The exterior and first‐floor foyer situate users within a university campus environment rather than an abstract laboratory setting. The laboratory entrance view emphasizes that aDNA research begins before entering the cleanroom, highlighting contamination‐prevention measures such as donning gloves and dedicated footwear prior to entering the laboratory. The Gowning Room introduces additional personal protective equipment (PPE), including hairnets, face masks, full Tyvek suits, and a second set of gloves, which together provide a barrier between the researchers and the aDNA samples. Users then progress through the three Inner Chambers, which represent the primary stages of aDNA laboratory work: sample preparation (Inner Chamber 1), aDNA extraction (Inner Chamber 2), and library preparation, which prepares the extracted DNA for sequencing (Inner Chamber 3). The tour concludes in a separate building that houses the Center for Genome Innovation sequencing center, where users are introduced to the instruments used to generate sequence data from aDNA extracts.

**FIGURE 3:**
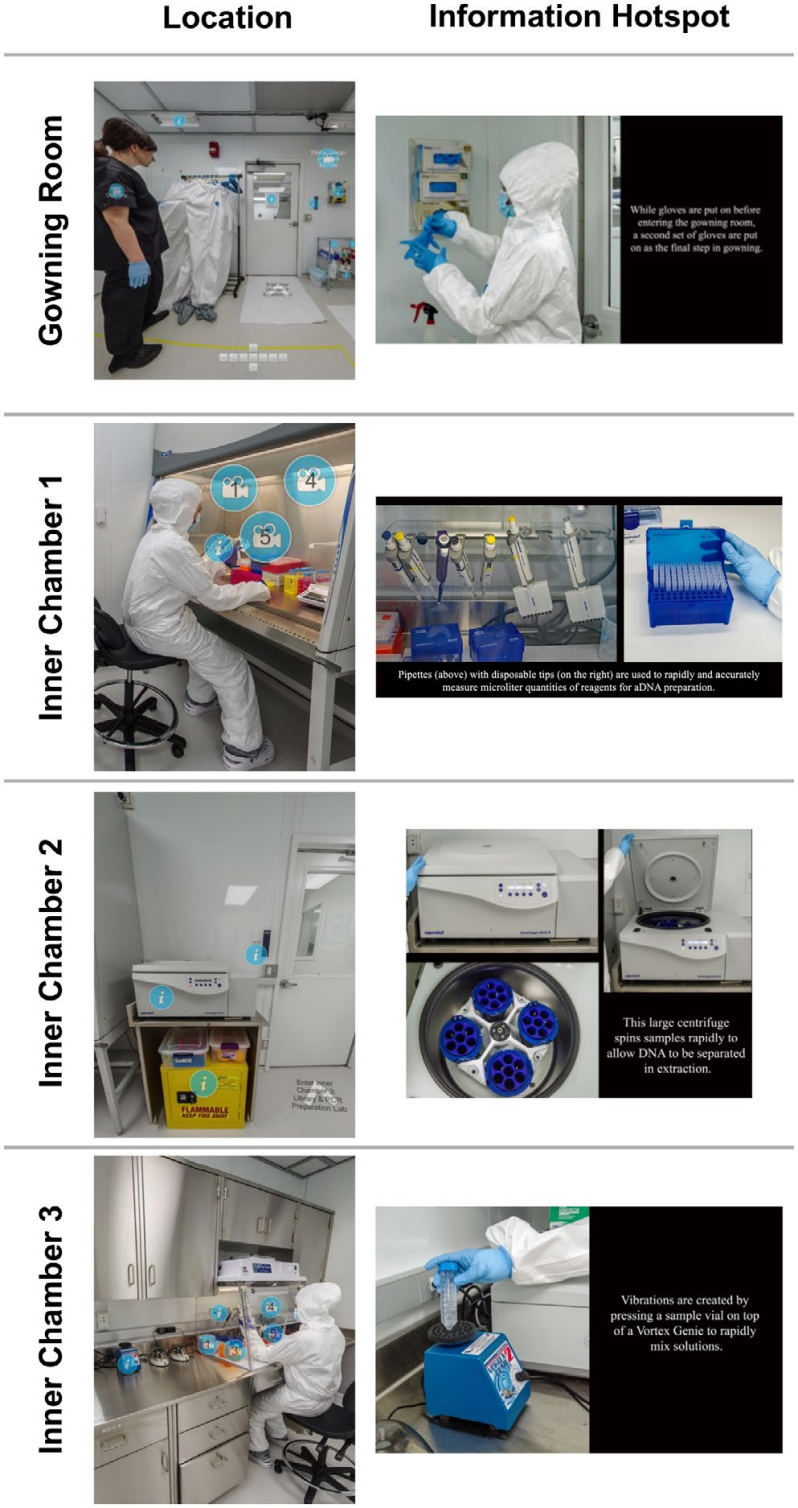
Examples of Information Hotspots and their locations in the four main chambers of the 360° Tour.

Within the six views spanning the Ancient DNA Laboratory entrance to the Center for Genome Innovation, the tour incorporates interactive components designed to provide users with information about the aDNA testing workflows. The main locations of the tour include Orientation Audio (Table 1) that automatically plays upon entry and provides key contextual information about the space. Information Hotspots (Figure 3, Table 2) appear as clickable icons that provide up close views of tools, instruments, and other equipment, often accompanied by brief explanatory text. Embedded Skills Videos (Table 3) present step‐by‐step demonstrations of key stages in the aDNA testing process. Lastly, a navigational schematic at the top right corner of the interface shows the layout of the four laboratory rooms to allow users to track their location as they move between spaces.

### 3.1 Detailed Description of Individual Spaces Within the 360° Tour

Here we detail the eight views of the 360° Tour to provide a written overview of the spaces showcased in the tour. Within each space, we highlight what users can observe and interact with as part of the tour.

#### 3.1.1 Exterior of Beach Hall at UConn

The first view presents the outside of Beach Hall, where the Ancient DNA Laboratory is located. The panoramic scene includes a red brick building, surrounding walkways, trees, and the campus landscape. An animated arrow labeled “Enter Beach Hall U. of Conn.” guides users into the building to continue the tour.

#### 3.1.2 Foyer on the First Floor of Beach Hall

Upon entering Beach Hall, users see the interior of the building showing the stairwell leading up to the Ancient DNA Laboratory. The scene includes a brown and beige mosaic tiled floor with a metal UConn seal in the center, an octagonal light fixture surrounded by painted grape vines on the ceiling, geological specimens from the Department of Geology on display, and signage for the College of Liberal Arts and Sciences at UConn. Doors and hallways to other labs and offices are also seen. Users proceed by selecting an animated arrow directing them towards three beige doors labeled “The stairs to the aDNA Lab” to enter the next view.

#### 3.1.3 Exterior of the Ancient DNA Laboratory

This view presents the preparation area and door to the Ancient DNA Laboratory. Orientation Audio plays to introduce the space (Table 1). There are four Information Hotspots that identify the materials required to enter the Gowning Room, which include a shoe rack with black crocs, a pack of sterile gloves, a bottle *DNA Away*, and an entry checklist that includes a list of to‐dos before entering the laboratory (Table 2). One Embedded Skill Video is shown, which features Bolnick describing the research that takes place in the laboratory (Table 3). Users click on an animated arrow pointing towards the lab's door labeled “Enter aDNA Lab” to enter the next view.

#### 3.1.4 Gowning Room

The Gowning Room antechamber serves as the entry point into the cleanroom laboratory. Upon entering, Orientation Audio describes the purpose of the room and its role in contamination prevention (Table 1). The space shows doors to the Inner Chambers 1 and 2, as well as a rack of white Tyvex suits, shoe storage, laboratory supplies, and waste containers. Seventeen Information Hotspots are present, of which six provide expanded descriptions when clicked, including about the overhead UV lights, sterile gloves, and bleach, ethanol, and *DNA Away* solutions for decontamination (Table 2). Three Embedded Skills Videos are included, where Fleskes demonstrates the gowning process and discusses aDNA research at Jamestown and ethical practices in aDNA research (Table 3). Animated navigational arrows titled “Enter Inner Chamber 1: Sample Preparation Lab” prompt the user to move into Inner Chamber 1.

#### 3.1.5 Inner Chamber 1 (Sample Preparation Lab)

This chamber is where bone and teeth samples are prepared for DNA extraction. Orientation Audio plays to introduce the purpose of the space (Table 1). Upon entering, stainless steel counters, cabinets, and a sink can be seen. As users pan throughout the room, they can see a yellow emergency shower station and a biosafety cabinet, where a researcher (Fleskes) is shown dressed in a full Tyvek suit working with a bovine bone within the cabinet. There are three Embedded Skills Videos (Table 3) demonstrating key parts of the sample preparation process, and eight Information Hotspots (Table 2) that highlight key tools such as the laminar flow hood, microbalance, hazardous waste container, and eyewash/shower station. To move to Chamber 2, users click the animated arrow labeled “Exit Inner Chamber 1: Sample Preparation Lab” to re‐enter the Gowning Room, where they then can click the animated arrow labeled “Enter Chamber 2: Extraction Lab” to continue the tour.

#### 3.1.6 Inner Chamber 2 (Extraction Lab)

Inner Chamber 2 is where DNA is extracted from samples that were prepared in Inner Chamber 1. Orientation Audio introduces the purpose of this space to the user (Table 1). The room shows a researcher (Fleskes) working at a laminar flow hood, as well as another emergency shower station, a sink, stainless steel cabinets, and counters with equipment such as a heating block, UV crosslinker, vortexer, and pipettes. Also shown are a freezer, a large centrifuge, and yellow and blue storage cabinets to store flammables and acids, respectively. Thirteen Information Hotspots (Table 2) and five Embedded Skills Videos (Table 3) document key steps of the aDNA extraction process. Users then click on the animated arrow labeled “Enter Inner Chamber 3: Library & PCR Preparation Lab” to proceed to the next stage of the tour. Doors and an animated arrow leading back to the Gowning Room are also displayed to allow users to move back to the antechamber if desired.

#### 3.1.7 Inner Chamber 3 (Library & PCR Preparation Lab)

Following DNA extraction, DNA samples are prepared for next generation sequencing through a process called library preparation. Orientation Audio plays upon entering (Table 1). The user can see stainless steel cabinets and a researcher (Fleskes) working at a biosafety cabinet. Panning around the room, five Information Hotspots highlight additional equipment such as centrifuges, a vortexer, and freezers (Table 2). Five Embedded Skills Videos demonstrate key steps in the library preparation process, including DNA damage repair, adapter ligation, and index polymerase chain reaction (PCR) preparation (Table 3). Users can then click on the white arrow labeled “Step 6: Library Preparation is complete. Off to the Modern Lab for PCR and Sequencing” to move to the last stage of the tour.

#### 3.1.8 Sequencing Center at UConn

The final view shows UConn's Center for Genome Innovation sequencing center, where DNA libraries are sequenced. Orientation Audio introduces the facility and its role in sequencing data generation (Table 1). The space includes Illumina sequencing instruments and computers, with two researchers (Fleskes and Bolnick) standing at a large white Illumina NovaSeq 6000 sequencing machine. One Information Hotspot describes the Illumina MiSeq sequencing machine and its function in imaging nucleotide bases to generate DNA sequencing data (Table 2). An Embedded Skills Video explains the DNA sequencing process and the types of research questions that can be addressed through aDNA analyses (Table 3). Users may return to the beginning of the tour by clicking the white navigation arrow labeled, “Return to Beach Hall.”

## 4 Context and Implementation

The 360° Tour developed out of a collaboration between anthropological geneticists Fleskes and Bolnick and the Jamestown Rediscovery Foundation, which is a nonprofit organization dedicated to the preservation and stewardship of Historic Jamestowne, the site of the first successful English colony in North America. Important to the organization's mission is continuing archaeological research grounded in public education. The 1607 Burial Ground Project was developed with this ethos, with the goal of using state of the art paleogenomics methods to understand the histories of the first colonists who passed away at Jamestown. Previously, Jamestown Rediscovery had utilized 360° VR technology to create an online tour of their onsite Archaerium Museum, which showcases artifacts recovered from the James Fort and the surrounding area to tell the story of Jamestown.^2^ Creating a similar 360° Tour of the Ancient DNA Laboratory at UConn was developed to facilitate online public engagement about the ongoing aDNA testing, supporting the organization's mission for public outreach and the researchers' goals for increasing transparency in the aDNA research process.

The 360° Tour was posted on the Jamestown Rediscovery Foundation's Historic Jamestowne website^1^ in early July 2025 with a comment box for users to provide optional feedback. Within the first 8 months of being online, the tour received 3090 views (early July 2025–early March 2026). All feedback received from the online comment box was positive and extended into inquiries regarding DNA testing to determine their possible relatedness to the Ancestors.

Beyond Jamestown's Bioarchaeology Program, the 360° Tour has been used for other community engagement research and educational purposes. Fleskes and Bolnick have separately referenced the 360° Tour as a part of community outreach efforts in the Chesapeake Bay region and throughout Texas, respectively. As an educational tool, Fleskes has utilized the tour in undergraduate lectures on aDNA and methods in archaeological science to increase student comprehension of paleogenomics. Specifically, it has been successfully implemented into the Introduction to Biological Anthropology, Introduction to Archaeology, and Human Biological Diversity classes at Dartmouth College. Thus, the tour provides diverse audiences with a glimpse into previously inaccessible research processes.

## 5 Discussion

Expanding scientific conversations to audiences outside of academia remains a persistent challenge, particularly for research fields that rely on controlled‐access laboratory infrastructure and highly technical workflows. As Dunnwald and colleagues observe, “scientists have struggled to make connections with the public to communicate what benefits [research] can provide” (2025, 1440). Virtual technologies offer one potential response to this challenge by facilitating interactive and immersive educational experiences that can reduce knowledge gaps between researchers and the public (Romero‐Luis et al. 2025). The 360° Tour contributes to these efforts by providing online access to a paleogenomics laboratory and by visualizing protocols involved in the generation of aDNA data, thereby increasing transparency around how knowledge in this field is produced.

In particular, an especially important contribution of the 360° Tour is its capacity to increase access to research environments that are otherwise inaccessible (Chong et al. 2021). Paleogenomic cleanroom laboratories are controlled‐access spaces designed to minimize contamination risk, which limits opportunities for direct engagement with descendant communities and the broader public in the spaces where the work is conducted. The use of VR technology has been recognized by other related fields, including genetics and biomedical research, as a means to increase accessibility without compromising environmental integrity. A growing number of laboratories have developed virtual lab tours to make research spaces visible to nonspecialist audiences, including the Wistar Institute,^3^ University of Massachusetts Chan Medical Genetics Laboratory,^4^ and Biology Labs at Arizona State University.^5^

Comparable uses of immersive visualization approaches have been widely adopted in cultural heritage sites and museums contexts, where virtual technologies allow audiences to experience cultural sites and collections that are inaccessible due to environmental conditions or conservation concerns (Bachiller et al. 2023; Banfi et al. 2023; Kyrlitsias et al. 2020). For example, the archaeological sites of Capo Colonna and Cala Minnola in South Italy are now submerged and can largely only be experienced through virtual technology (Bruno et al. 2018). Similarly, physical, economic, or other barriers may also limit access to cultural heritage sites. The Zamani Project has worked to expand public access to Petra, Jordan through a virtual walk‐through created to bring these sites to a wider audience (Wessels et al. 2014). Notably, immersive VR applications have been shown to “[increase] the participants' sense of presence…which leads to higher engagement in the experience, motivation, and cognitive processing of the material” (Kyrlitsias et al. 2020, 8). In this respect, the virtual technologies employed in the 360° Tour align with broader efforts in cultural heritage studies to use visual technology as a means of enhancing accessibility and comprehension.

Despite these developments, virtual technologies remain underutilized in paleogenomics. Existing efforts primarily consist of videos, including recordings of academic presentations,^6^ community engagement or public outreach presentations (Zolik et al. 2023),^7^ ^,^ ^8^ ^,^ ^9^ paleogenomic methods,^10^ ^,^ ^11^ and aDNA laboratory spaces, including from the Stone Lab at Arizona State University,^12^ the Ancient DNA Lab at Uppsala University,^13^ and the UC Santa Cruz Paleogenomics Laboratory.^14^ More recently, Fleskes and colleagues documented the aDNA extraction process using a GoPro for community engagement purposes as part of the Anson Street African Burial Ground Project (2025). The 360° Tour complements and extends these efforts by integrating stepwise video demonstrations of laboratory workflows within a virtual environment to offer a more comprehensive platform. Together, these initiatives represent important initial steps towards increasing accessibility surrounding the paleogenomics research process.

### 5.1 Ethical Implications for Paleogenomics Research

Beyond outreach, increased accessibility to laboratory spaces has important ethical implications for paleogenomics research. Research involving human ancestral remains raises distinct ethical challenges related to informed consent and governance of genetic data (Gibbon et al. 2024; Kaestle and Horsburgh 2002; Wagner et al. 2020). While Institutional Review Boards require informed consent from living human research participants, deceased archaeological individuals fall outside of these protections. As human Ancestors are unable to advocate for themselves, researchers must navigate complex questions regarding ethical and methodological decision‐making (Turner et al. 2018). Some scholars argue that informed proxy consent may be obtained from descendant communities or connected communities of care (Gibbon et al. 2024; Kaestle and Horsburgh 2002). In such cases, it is essential that the communities acting on behalf of the deceased are provided with clear and comprehensive information about research practices and protocols, so that they can make well‐informed decisions about the conduct of the research and the subsequent handling of the resulting data. In these contexts, limited visibility and understanding of laboratory processes can exacerbate existing power asymmetries between researchers and descendant communities or other communities of care (Ávila‐Arcos et al. 2022; Gibbon et al. 2024; Yáñez et al. 2023). Tools that increase transparency therefore play a critical role in supporting ethical research practices by enabling communities to better understand how aDNA analyses are conducted.

In addition to informed consent, transparency is central to ongoing discussions of data sovereignty within paleogenomics. Data sovereignty frameworks emphasize the rights of communities to retain authority over how genetic data derived from their Ancestors are generated, interpreted, shared, and reused (de la Cova et al. 2024; Gilmore et al. 2024; Tamburrini et al. 2023). These principles are increasingly articulated through models such as “DNA‐on‐loan,” in which researchers act as stewards rather than owners of genetic material and associated data (Arbour and Cook 2006; Ávila‐Arcos et al. 2022). Visualization of destructive sampling procedures, laboratory environments, and research equipment through virtual technologies can support these aims by enabling communities to engage more fully in decisions surrounding data governance, interpretation, and long‐term stewardship. Such transparency facilitates more equitable collaboration between scientists and communities, and strengthens trust in paleogenomic research practices (Ávila‐Arcos et al. 2022; de la Cova et al. 2024; Judd and McKinnon 2021; Tamburrini et al. 2023).

More broadly, expanding access to research spaces also has implications for equity and inclusion in science. Paleogenomics—like other STEM fields—benefits from diversification, with different perspectives strengthening scientific progress through novel questions asked and unique interpretations of results produced (Bolnick et al. 2019; Schroeder 2022). Antón and colleagues argue that a key barrier to recruiting a more diverse cohort of researchers in STEM and Biological Anthropology is widespread public misperception about “what scientists do and who scientists are” (2018, 167). Virtual technologies that immerse users in scientific experiences can offer a direct means of making scientific work more visible and approachable. Within paleogenomics, effective science communication is particularly important for addressing research misconceptions and for ethical engagement with marginalized communities. Lemke and colleagues explain that within “the field of human genetics and genomics, instances of oppression and exclusion such as research abuses and eugenic sterilization have led to mistrust among populations that have been, and continue to be, marginalized and underrepresented in research” (2022, 1565). Because paleogenomics emerges from this history, it must take intentional steps towards ethically informed inclusion through practices such as benefit sharing and transparent communication of findings (Lemke et al. 2022). Virtual reality technologies provide one pathway for bridging relationships between researchers and populations historically underrepresented within STEM fields to support the development of a more diverse generation of scientists that will ultimately advance the field as a whole.

## 6 Conclusion and Future Directions

This study demonstrates how immersive VR technologies can be used to increase accessibility and transparency within controlled‐access research environments, such as paleogenomic cleanroom laboratories. The 360° Tour aims to provide a structured and accessible representation of laboratory workflows that are typically difficult for nonspecialist audiences to observe and comprehend. In addition, as paleogenomics continues to engage with questions of informed consent, proxy consent, and data sovereignty, tools that enhance understanding of laboratory practices can support more equitable and informed participation by descendant communities and other communities of care. In this way, the 360° Tour contributes to broader efforts to demystify scientific research processes and to make the production of paleogenomic knowledge more visible to diverse stakeholders, supporting more transparent research practices.

The 360° Tour is intended to remain an online resource on the Jamestown Rediscovery Foundation's Historic Jamestowne website and be used for education and outreach initiatives for the 1607 Burial Ground Project. In the future, the tour could be incorporated into a physical museum exhibit at the Jamestown Rediscovery Foundation's Archaerium Museum to expand online accessibility. Future iterations of the tour may incorporate additional interactive elements, such as guided activities, 3D models, or exploratory tasks, to further enhance audience engagement and learning (Levonis et al. 2021).

More broadly, this work highlights the need for greater scholarly attention to the development, evaluation, and publication of tools for public science communication within paleogenomics and related fields in ecology and evolution. Despite growing recognition of the importance of transparency, accessibility, and ethical engagement (Bolnick et al. 2019; Fleskes et al. 2022; Kowal et al. 2023; National Academies of Sciences, Engineering, and Medicine 2017), such tools are not often described in peer‐reviewed literature. Publishing and critically assessing these approaches will help establish shared standards, evaluation methods, and facilitate broader adoption of these tools. We therefore encourage other researchers working in paleogenomics and allied disciplines to develop and describe similar tools in an effort to recognize science communication infrastructure as an integral component of ethical research practice.

## Author Contributions

**Christina M. Balentine:** conceptualization (equal), visualization (supporting), writing – original draft (lead), writing – review and editing (lead). **Alisha Khalil:** conceptualization (equal), funding acquisition (supporting), writing – original draft (supporting), writing – review and editing (supporting). **Abigail Sink:** writing – original draft (supporting), writing – review and editing (equal). **Horvey Palacios:** writing – original draft (supporting). **Deborah A. Bolnick:** conceptualization (equal), resources (lead), supervision (equal), writing – review and editing (equal). **Michael Lavin:** conceptualization (equal), supervision (equal), writing – review and editing (supporting). **Dave Givens:** conceptualization (equal), supervision (equal), writing – review and editing (supporting). **Chuck Durfor:** conceptualization (lead), data curation (lead), software (lead), visualization (lead), writing – original draft (supporting), writing – review and editing (supporting). **Raquel E. Fleskes:** conceptualization (lead), funding acquisition (lead), project administration (lead), supervision (equal), writing – original draft (lead), writing – review and editing (lead).

## Funding

This project was supported by the Dartmouth College Undergraduate Research Assistantship and National Science Foundation (REF & DAB, #2105384).

## Conflicts of Interest

The authors declare no conflicts of interest.

## Supporting information


**Appendix S1:** Full textual description of the 360° tour.

## Data Availability

Data sharing not applicable—no new data generated, or the article describes entirely theoretical research.

## Endnotes

^1^

https://historicjamestowne.org/uconn‐adna‐lab‐tour/.

^2^

https://historicjamestowne.org/archaearium‐virtual‐tour‐2/.

^3^

https://www.wistar.org/about‐wistar/tour‐wistar‐labs/.

^4^

https://www.umassmed.edu/es/muellerlab/technology/virtual‐tour‐of‐lab/.

^5^

https://askabiologist.asu.edu/explore/virtual‐360‐laboratories.

^6^

https://youtu.be/NcEqO6yx68c?si=IMzOjvq_Eu3cjm4I.

^7^

https://youtu.be/5ZKIKLHhHNg?si=W94YKCm2CyIOuvxL.

^8^

https://youtu.be/c2ppreiB1PQ?si=dhAveGBGb2‐vGtjz.

^9^

https://youtu.be/lRXZjv7iM5k?si=3KntDS_K6aD8wlU3.

^10^

https://www.youtube.com/watch?v=48DFUBwr9dk.

^11^

https://www.youtube.com/watch?v=Mviy0wGett4.

^12^

https://www.youtube.com/watch?v=gp_yKX9JhcI.

^13^

https://youtu.be/6ZU0lHuGfhA?si=‐‐tSQg3ZrRSwv55y.

^14^

https://www.youtube.com/watch?v=KTp8T4miOzM&t=24s.
