# Supplementary material for: Virtual Reality for Outreach and Community‐Engagement in Paleogenomics: Development of a 360° Tour of an Ancient DNA Laboratory
Source: Ecol Evol. 2026 Jul 17;16(7):e74033. doi: 10.1002/ece3.74033 (PMC13379311; doi:10.1002/ece3.74033)
Supplement: Supplementary file 2 — Appendix S1: Full textual description of the 360° tour. [file ECE3-16-e74033-s001.docx]

**APPENDIX**

**Full Textual Description of the 360° Tour**

**View 1 Exterior of Beach Hall at UConn**

***View 1 Description:*** The first view presents the outside of Beach Hall, where the Ancient DNA Laboratory is located. The panoramic scene includes a red brick building, surrounding walkways, trees, and the campus landscape. An animated arrow labeled ‘Enter Beach Hall U. of Conn.’ guides users into the building to continue the tour.

***View 1 Orientation Audio:*** N/A

***View 1 Information Hotspots:*** N/A

***View 1 Embedded Skills Videos Transcripts:*** N/A

**View 2 Foyer on the first floor of Beach Hall**

***View 2 Description:*** Upon entering Beach Hall, users see the interior of the building showing the stairwell leading up to the Ancient DNA Laboratory. The scene includes brown and beige mosaic tiled floor with a metal UConn seal in the center, an octagonal light fixture surrounded by painted grape vines on the ceiling, geological specimens from the Department of Geology on display, and signage for the College of Liberal Arts and Sciences at UConn. Doors and hallways to other labs and offices are also seen. Users proceed by selecting an animated arrow directing them towards three beige doors labeled ‘The stairs to the aDNA Lab’ to enter the next view.

***View 2 Orientation Audio:*** N/A

***View 2 Information Hotspots:*** N/A

***View 2 Embedded Skills Videos Transcripts:*** N/A

**View 3 Exterior of the Ancient DNA Laboratory**

***View 3 Description:*** This view presents the preparation area and door to the Ancient DNA Laboratory. Orientation Audio plays to introduce the space. There are four Information Hotspots that identify the materials required to enter the Gowning Room, which include a shoe rack with black crocs, a pack of sterile gloves, a bottle *DNA Away*, and an entry checklist that includes a list of to-dos before entering the laboratory. One Embedded Skill Video is shown, which features Bolnick describing the research that takes place in the laboratory. Users click on an animated arrow pointing towards the lab’s door labeled ‘Enter aDNA Lab’ to enter the next view.

***View 3 Orientation Audio:*** *“Welcome to the Ancient DNA Lab at the University of Connecticut. This is the outside of our lab space, where we prepared to enter into the inner gowning room.”*

***View 3 Information Hotspots:***

| Information Hotspots | Accompanying Descriptive Text |
| --- | --- |
| 1: Shoe Rack and Black Crocs | *“To reduce possible contamination, street shoes are removed and Black Lab Crocs are worn”* |
| 2: A Pack of Sterile Gloves | *“Before you enter the lab, you put on a pair of sterile gloves”* |
| 3: A Bottle of *DNA Away* | *“Once Black Lab Crocs and gloves are put on, contaminants on the gloves are removed by DNA AWAY, (a dilute alkaline solution) that removes DNA from equipment that would be damaged by a 10% bleach solution”* |
| 4: Entry Checklist | *-* |

***View 3 Embedded Skills Videos Transcripts:***

Video 1 aDNA Overview

Hi, my name is Deborah Bolnick. I am a professor of anthropology at the University of Connecticut. I am an anthropological geneticist and a biocultural anthropologist.

In our lab, we’re interested in exploring how sociopolitical forces, historical events, and social inequality shape human genomic diversity. We study DNA from ancient and contemporary peoples and are interested in how we can use DNA, in conjunction with other kinds of evidence, to help reconstruct population histories in the Americas. In our lab, we’re especially interested in the genetic impacts of settler colonialism and how historical events over the last few hundred years have affected Indigenous communities, European settlers, and individuals of African descent who were brought here through the transatlantic slave trade.

In this work, we work closely with a variety of other research partners. We collaborate with archeologists, with Indigenous community members, historians, genealogists, and many others. In this work, we’re also very interested in the social, legal, and ethical implications of this research and what impact genomic research can have for members of living communities today.

**View 4 Gowning Room**

***View 4 Description:*** The Gowning Room antechamber serves as the entry point into the cleanroom laboratory. Upon entering, Orientation Audio describes the purpose of the room and its role in contamination prevention . The space shows doors to the Inner Chambers 1 and 2, as well as a rack of white Tyvex suits, shoe storage, laboratory supplies, and waste containers. Seventeen Information Hotspots are present, of which six provide expanded descriptions when clicked, including about the overhead UV lights, sterile gloves, and bleach, ethanol, and *DNA Away* solutions for decontamination. Three Embedded Skills Videos are included, where Fleskes demonstrates the gowning process, and discusses aDNA research at Jamestown and ethical practices in aDNA research. Animated navigational arrows titled ‘Enter Inner Chamber 1: Sample Preparation Lab’ prompt the user to move into Inner Chamber 1.

***View 4 Orientation Audio:*** *“This is the gowning room of the Ancient DNA Lab. This room serves as the halfway point, where we put on personal protective equipment, such as gloves and Tyvek suits, in order to enter into the inner suites.”*

***View 4 Information Hotspots:***

| Information Hotspots | Accompanying Descriptive Text |
| --- | --- |
| 1: Shoe Rack and White Crocs | *“The Black Lab Crocs are removed during gowning and replaced by White Lab Crocs to enter the inner chambers”* |
| 2: UV Lights | *“These lights prevent any modern DNA from being amplified to help prevent contamination”* |
| 3: Sterile Gloves | *“While gloves are put on before entering the gowning room, a second set of gloves are put on as the final step in gowning”* |
| 4: 10% Bleach | *“A 10% Bleach Solution is frequently sprayed on gloves to remove DNA contamination”* |
| 5: 70% Ethanol | *“A 70% Ethanol Solution is sprayed on gloves to remove the 10% Bleach Solution”* |
| 6: *DNA Away* | *“ DNA Away (a dilute alkaline solution) removes DNA contamination from tools that cannot withstand bleach.”* |
| 7–8: Inner Chamber 1 Doorplate & Checklist | *-* |
| 9–10: Inner Chamber 2 Doorplate & Checklist | *-* |
| 11: Laboratory Safety Card | *-* |
| 12: BSL1 Card | *-* |
| 13: Storage Drawers for Masks, Hairnets | *-* |
| 14: Storage Drawers for Pipettes and Pipette Tips | *-* |
| 15: Storage Drawers for Misc. Lab Supplies | *-* |
| 16: Storage Drawers for Kimwipes, Spray Bottles | *-* |
| 17: Checklist for Leaving the Lab | *-* |

***View 4 Embedded Skills Videos Transcripts:***

Video 1 aDNA Research at Jamestown

Hi, my name is Dr. Raquel Fleskes. I’m currently a postdoc at the University of Connecticut. I do work in ancient DNA. Ancient DNA is the study and extraction of DNA from archaeological materials. I’m here today in our ancient DNA lab. We have to carry out this work in a special lab that helps us keep our samples free from any modern DNA, and that’s because contamination is really important in ancient DNA. We have to really work hard to make sure that our samples are not contaminated from DNA from living individuals such as myself, the researcher. So, we’re really excited to be here on the forefront of research in ancient DNA in the colonial time period. Ancient DNA provides a window into understanding the past, understanding things like ancestry or where people come from; relatedness, how people are related to each other, how burials are related to each other; as well as things like phenotype and selection.

And we’re able to do this by sequencing and extracting the DNA from archaeological individuals. Ancient DNA also helps us think and dive a little bit more deeply into bioarchaeological research. So, work with other researchers such as archaeologists and osteologists help build a context for who these people are, what were their lives like.

Ancient DNA can come in and also provide a complementary perspective by thinking about things like ancestry and relatedness. So, we’re really excited to be here and working with Jamestown on some really incredibly exciting research on the remains of individuals interred here.

Something that’s also really important in doing ancient DNA research and really any research on archaeological populations is community engagement. And that’s because while we’re working with populations in the past, we’re doing it in today’s moment. And there are communities, descendants, and living individuals that connect to the ancestors, these archaeological individuals that we are researching. So, part of the initiative with the Bioarchaeological Research Program at Jamestown is to make sure that we’re doing this work in full transparency as much as possible to remove the black box and work with communities to understand how the science is being carried out and why this information is important. And also, to answer questions that folks are interested in understanding. We hope to learn things like, where do people come from, what their lives were like. So please stay tuned for the space to learn more about ancient DNA at Jamestown.

Video 2 Ethical Design of aDNA Studies

Now before beginning any ancient DNA project, it’s very important to make sure you have proper ethical permissions to conduct the study. Most important in this is contacting and being in constant communication with potential descendant communities or communities that identify with the archaeological ancestors that are being studied. This can be a spiritual connection, a direct descendant connection, or even a geographical or cultural connection for residents living in the same area as the archaeological ancestors were found.

Now part of the study design in close collaboration with descendant communities or community stakeholders is deciding how destructive we want these destructive processes of ancient DNA to be. Now it’s no secret that ancient DNA is inherently a destructive process. So this means that we would take parts of archaeological ancestors, their skeletal remains—such as a tooth fragment or, for instance, a petrous bone, which is the bone that surrounds your inner ear—and we take these bones and we grind them up into small fragments to create a powder that then we use for DNA extraction. However, in the last 10 years and more recently in the last couple of years, new protocols have been developed which allow us to take a less invasive approach for DNA. This is what we call a minimally invasive extraction protocol. These types of protocols involve taking an intact tooth with a good root structure and soaking it in an extraction buffer for a number of hours. And what we’re targeting is that DNA in the cementum of the tooth or around the outside of the tooth. So, this results in a slightly cleaner appearance of the tooth, but does not involve any direct drilling, so thus it’s minimally invasive. We’re able to get pretty comparable results using these minimally invasive methods as compared with the fully destructive ones.

So here at the University of Connecticut, we practice both destructive and minimally destructive or minimally invasive methods to fit the needs of the communities that we work with. And important in continuing this conversation is having conversations around data access, what happens to the genomic data, what types of questions communities are interested in understanding.

Video 3 PPE Gowning

A surgical mask reduced exhaled DNA contamination. A hairnet is worn to reduce DNA contamination from falling pieces of hair. A Tyvek suit is worn to reduce contamination from DNA on loose skin, hair, or perspiration. The black crocs are replaced with white crocks that remain in the cleanroom. Sleeves that extend from the elbow to the gloves are worn to further reduce contamination. A second pair of gloves are worn to further reduce contamination. Gloves are sprayed with 10% bleach and 70% ethanol to remove further contamination. Good to go into the inner aDNA lab suites.

**View 5 Inner Chamber 1 (Sample Preparation Lab)**

***View 5 Description:*** This chamber is where bone and teeth samples are prepared for DNA extraction. Orientation Audio plays to introduce the purpose of the space. Upon entering, stainless steel counters, cabinets, and a sink can be seen. As users pan throughout the room, they can see a yellow emergency shower station and a biosafety cabinet, where a researcher (Fleskes) is shown dressed in a full Tyvek suit working with a bovine bone within the cabinet. There are three Embedded Skills Videos demonstrating key parts of the sample preparation process, and eight Information Hotspots that highlight key tools such as the laminar flow hood, microbalance, hazardous waste container, and eyewash/shower station. To move to Chamber 2, users click the animated arrow labeled ‘Exit Inner Chamber 1: Sample Preparation Lab’ to re-enter the Gowning Room, where they then can click the animated arrow labeled ‘Enter Chamber 2: Extraction Lab’ to continue the tour.

***View 5 Orientation Audio:*** *“You have entered into the first inner chamber of the Ancient DNA Lab. This is the sample preparation suite. In this suite we prepare bone or teeth samples for DNA extraction.”*

***View 5 Information Hotspots:***

| Information Hotspots | Accompanying Descriptive Text |
| --- | --- |
| 1: Laminar Flow Hood | *“A laminar flow hood protects against dust and other potential contaminants via a constant, unidirectional flow of air inside the hood. It also reduces the worker’s exposure to harmful materials used inside the hood”* |
| 2: Microbalance | *“The weight of bone powder samples and reagents is measured with a Microbalance”* |
| 3: Bone Dust Tools | *“These tools are used to turn pieces of bone into bone dust”* |
| 4: Hazardous Waste Container | *“Careful disposal of hazardous waste is critical for lab safety and high quality study results”* |
| 5: Cleaning Instructions | *-* |
| 6–7: Eyewash/Shower Station & Sign | *“In case of an accident, the eyewash provides a rapid method of flushing harmful chemicals from the eyes”* |
| 8: Checklist for Leaving Inner Chamber 1 | *-* |

***View 5 Embedded Skills Videos Transcripts:***

Video 1 Decontamination Using UV Crosslinker

*{Video begins with image of a UV crosslinker, which is a rectangular box like a toaster oven. Accompanying the image is a caption: “Within the UV Crosslinker, supplies and sample vials are decontaminated using high intensity UV light.”}*

Alright, so the first step in this process is to clean everything that could possibly get in contact with the bone. So, all of these supplies that might be used for bone sampling. We put them into a UV crosslinker. And while that’s cooking, we’re going to then soak our Dremel supplies in a 10% bleach solution for 30 seconds. Next, we’re going to rinse it, dump the bleach out, and then we’re going to add 70% ethanol. This is going to basically remove all of the bleach residue. Okay, and I’m going to dump these out. And then we’re going to wipe them down. Okay. Alright, and these should be done. And we’ll pull these out.

Video 2 Preparing Bone Samples for aDNA Processing Part 1

*{Video begins with an image of a Dremel handheld drill inside a biosafety hood, with the following caption: “In this small enclosed space called a Biosafety Hood, small pieces of bone are sectioned off using a Dremel rotary tool.”}*

So, we’ve loaded in our supplies for drilling. We’re going to target this cow tibia as an example. The good areas for DNA are this really dense cortical bone here and not this kind of spongy trabecular bone. So, what we’re going to use, is with this Dremel, is section off a small portion of this dense cortical bone.

While we’re watching the section of bone being removed from the larger cow tibia, we can discuss other samples of DNA that are often used for DNA analysis. For example, tooth roots have been shown to harbor rich sources of DNA. In addition, the petrous portion of the temporal bone, or the bone that surrounds your inner ear, contains some of the densest bone in the human body. The denser the bone, the better it is for DNA preservation, making it an optimal source for many ancient DNA studies. Now off camera, we’re going to finish sectioning off this DNA sample.

Video 3 Preparing Bone Samples for aDNA Processing Part 2

*{Video begins with an image of a grinder mill, which has arms to hold metal vials that are shaken. Accompanying the image is a caption: “Pieces of bone and a stainless steel ball are placed in a grinder mill. While shaking the bone is crushed to dust.”}*

Okay, here we have a metal ball and places it in here into the grinder mill. And this ball is going to shake very fast, which will powder our sample. Ok, make them secure.

Alright, so now we’re going to check our mixer mill. Great! We’re going to now gently scrape the bone powder. You see, it’s really completely pulverized the bone. So, for aDNA extraction, we’re going to need approximately anywhere between 0.1 to 0.2 grams, which is very, very small. It’s like almost like a pinch of salt. So, we’re going to weigh this out and see if we have enough. So now we’re done with our sample preparation and we’re going to head into the extraction room to extract DNA.

**View 6 Inner Chamber 2 (Extraction Lab)**

***View 6 Description:*** Inner Chamber 2 is where DNA is extracted from samples that were prepared in Inner Chamber 1. Orientation Audio introduces the purpose of this space to the user. The room shows a researcher (Fleskes) working at a laminar flow hood, as well as another emergency shower station, a sink, stainless steel cabinets, and counters with equipment such as a heating block, UV crosslinker, vortexer, and pipettes. Also shown are a freezer, large centrifuge, and yellow and blue storage cabinets to store flammables and acids, respectively. Thirteen Information Hotspots and five Embedded Skills Videos document key steps of the aDNA extraction process. Users then click on the animated arrow labeled ‘Enter Inner Chamber 3: Library & PCR Preparation Lab’ to proceed to the next stage of the tour. Doors and an animated arrow leading back to the Gowning Room are also displayed to allow users to move back to the antechamber if desired.

***View 6 Orientation Audio:*** *“You are now in our second inner chamber of the Ancient DNA Lab. This is the DNA extraction suite. In this chamber, DNA extractions are conducted.”*

***View 6 Information Hotspots:***

| Information Hotspots | Accompanying Descriptive Text |
| --- | --- |
| 1: Pipettes | *“Pipettes (above) with disposable tips (on the right) are used to rapidly and accurately measure microliter quantities of reagents for aDNA preparation”* |
| 2: Large Centrifuge | *“This large centrifuge spins samples rapidly to allow DNA to be separated in extraction”* |
| 3: Flammable Storage Cabinet | *“Acid and Flammable Storage Cabinets provide a safe way to store dangerous materials”* |
| 4: Acid Storage Cabinet | *“Acid and Flammable Storage Cabinets provide a safe way to store dangerous materials”* |
| 5: Inner Chamber 3 Doorplate | *“Library & PCR preparation laboratory”* |
| 6: Heating Block | *“The heating block provides a controlled heated environment for sample preparation”* |
| 7: Checklist for Leaving Inner Chamber 2 | *-* |
| 8: Sink | *“Time to do the dishes with 10% Bleach and 70% ethanol. Lab dishes are soaked in the bucket in a 10% bleach solution for decontamination”* |
| 9: Vortexer | *“Vibrations created by pressing a sample vial on the top of a Vortex Genie rapidly mixes solutions”* |
| 10: UV Crosslinker | *“Within the UV Crosslinker, supplies and sample vials are decontaminated using high intensity UV light”* |
| 11: Cabinet of Reagents | *-* |
| 12–13: Eyewash/Shower Station & Sign | *“In case of an accident, the eyewash provides a rapid method of flushing harmful chemicals from the eyes”* |

***View 6 Embedded Skills Videos Transcripts:***

Video 1 Beginning DNA Extraction

Alright, so now we have prepared our bone sample and powdered it for DNA extraction. What we’re going to do now is actually begin the process of doing DNA extraction. This begins by first doing an extraction buffer, where we’re going to demineralize the bone over a period of 24 hours under heat in an incubator. So, what we’re going to do now is make that extraction buffer and then apply it to our bone powder. First, we’re going to do EDTA, which demineralizes the bone.

*{Image of pipettes and pipette tips with the following caption: “Pipettes (above) with disposable tips (on the right) are used to rapidly and accurately measure microliter quantities of reagents for aDNA preparation.”}*

Then we’re going to do proteinase K, which eats kind of the enzyme, you know, will eat all the extra proteins we don’t want. And then lastly is water. Alright, so we’ve now made our extraction buffer, and it has been UV’ed and it’s ready to go. So, we’re now going to add that extraction buffer to our sample tubes to begin the process of extraction. So here you can see me using a large pipetter to add in a little bit of our extraction buffer to our DNA sampling tubes. We will then carry these DNA sampling tubes to our incubator where they will sit at 56 degrees Celsius overnight.

*{Image of white rectangular incubator, with the following caption: “The HERATHERM Incubator provides a heated constant temperature environment for sample preparation.”}*

Video 2 DNA Incubation Part 1

*{Video begins with an image of white rectangular incubator, with the following caption: “The HERATHERM Incubator provides a heated constant temperature environment for sample preparation.”}*

The Heratherm Incubator provides a heated constant temperature environment for sample incubation. The incubator maintains samples at 56 degrees Celsius with a constant mixing by rotation. Here you can see me adding in our DNA samples for incubation. I place each of these tubes on a rotating mixer. This mixer will rotate 360 degrees to make sure our bone powder and extraction buffer make appropriate contact. This helps to increase the effectiveness of our incubation. Now we close the doors and the samples incubate.

Video 3 Making the Binding Buffer

So now that our DNA is in the incubator and is going through the process of DNA extraction, we need to prepare two more buffers which allow us to pull that DNA out of the solution and elute it. The first buffer we’re going to make is called a binding buffer. This is based with Guanidine Hydrochloride, which is a protein precipitate. And we’re going to basically weigh out these salts and add a bunch of other reagents to it, and then make our final TET buffer as well.

So, what I’m first going to do is weigh out 23 grams of Guanidine Hydrochloride. Okay, 23.8… perfect! Alright, now we have that, so now we’re going to do is add this to our binding buffer. Our binding buffer is full of salts. Now we’re going to add 25 microliters of Tween-20. And we’re going to now fill this with water, 30 mL. Basically, now it’s kind of dissolving the salt. Now we’re going to add 1.5 mL of sodium acetate and this just helps with the pH. Okay, so now we’re going to dissolve these salts. Okay. We’re looking good! So now what we’re going to do is now that we’ve dissolved our salts or mixed them up, we’re going to add isopropyl alcohol. And what this will do is really kind of help this get into solution. Alright, and now we have assembled our binding buffer.

Video 4 Spinning Down Samples in Centrifuge

*{Video begins with images of a tabletop centrifuge machine with the following caption: “The centrifuge gently precipitates material from solution by spinning at a high speed.”}*

Alright, so we’ve just now taken our DNA samples out of the incubator. The DNA has now been demineralized and pulled into the surrounding liquid. So, what we need to do now is spin down these tubes very, very fast so that the bone powder pellets at the bottom, forming a hard kind of rock pellet. How we’re going to do that is using a DNA centrifuge. I’m going to load these guys in, put the cap on, and we’re going to push start.

Video 5 DNA Isolation

So now we have our DNA that has been in the incubator and we have spun it down, so we’re ready now to add our DNA sample to our binding buffer that we’ve made before. What we’re going to do is… this is our binding buffer that we’ve made previously, and I’ve just poured it into a smaller tube. And what we’re going to do is pour a little bit of our sample, and the DNA is in liquid here, to this binding buffer and mix it.

Now our next step is going to be pouring our binding buffer, this contains our DNA, into our spin column. So this spin column here contains a special reservoir where we will pour our DNA binding buffer solution into here, which will then pass through this column, and the DNA will get stuck in this membrane. And all the rest of the solution will pass through this tube into the collection vial.

So now what we’re going to do is we’re going to spin these DNA samples through the Zymo column. We’re going to use our large centrifuge to do this. We’re going to place each one into this, let’s close it and I’ll push start.

So now we’ve spun through our DNA which has now, because of the binding buffer that we’ve made, stuck to this white filter. So, the DNA is stuck here, and all the water and other solutions have passed through. So, what we’re going to do is wash this filter, wash the DNA, using a PE buffer and this buffer will basically remove the salts, all the extra proteins, and everything else that we don’t want. We just want to elute, or release, the DNA that’s in this membrane right here.

So now we have our final release buffer. We have now purified and washed our DNA. Now we need a solution to say, “okay DNA, it’s time to go, it’s time to let go,” and this is what our TET buffer does. Our TET buffer has been sitting and incubating at 56 degrees Celsius which is the right temperature to allow that DNA to really get nice and loose, and released from the membrane. So, what we’re going to do is take this TET buffer into the hood and do our last DNA extraction step.

Okay, so now we’re ready to isolate our DNA from our membrane. So, what we’re going to do is remove this big tube because we don’t need it anymore. So, we’re going to unscrew it and place it into our smaller collection vial. Put it right in here, then we’re going to screw and break it off. So now we have our spin column, with our DNA that’s trapped in this membrane, in our new DNA collection tube. So, what we’re going to do now is put our final buffer which will release the DNA that’s trapped in here and have it go through to the bottom of the tube.

Okay so now we’ve added our elution buffer. What we need to do now is put it through another centrifuge to spin it through to say, “hey DNA, it’s time to release.” So, what we’re going to do is put it into this centrifuge, close the lid, and push start.

So now the DNA has been released from this white membrane here and is now found in the liquid solution right underneath. So, the last step that we have to do is to take out our spin column, throw it away, close our lid, and we are now finished with DNA extraction.

**View 7 Inner Chamber 3 (Library & PCR Preparation Lab)**

***View 7 Description:*** Following DNA extraction, DNA samples are prepared for next generation sequencing through a process called library preparation. Orientation Audio plays upon entering. The user can see stainless steel cabinets and a researcher (Fleskes) working at a biosafety cabinet. Panning around the room, five Information Hotspots highlight additional equipment such as centrifuges, a vortexer, and freezers. Five Embedded Skills Videos demonstrate key steps in the library preparation process, including DNA damage repair, adapter ligation, and index polymerase chain reaction (PCR) preparation. Users can then click on the white arrow labeled ‘Step 6: Library Preparation is complete. Off to the Modern Lab for PCR and Sequencing’ to move to the last stage of the tour.

***View 7 Orientation Audio:*** *“This is the third and final inner chamber of the Ancient DNA Lab. This is the library preparation suite. In this chamber, we take our extracted DNAs and prepare them for sequencing.”*

***View 7 Information Hotspots:***

| Information Hotspots | Accompanying Descriptive Text |
| --- | --- |
| 1: Vortexer | *“Vibrations created by pressing a sample vial on the top of a Vortex Genie rapidly mixes solutions”* |
| 2: Pipettes | *“Pipettes (above) with disposable tips (on the right) are used to rapidly and accurately measure microliter quantities of reagents for aDNA preparation”* |
| 3: Checklist for Leaving Inner Chamber 3 | *-* |
| 4: Centrifuge | *“The centrifuge gently precipitates material from solution by spinning at a high speed”* |
| 5: Freezer | *“Temperature-sensitive reagents and samples are stored in a -20°C freezer”* |

***View 7 Embedded Skills Videos Transcripts:***

Video 1 Removing Ancient DNA Damage – UDG Treatment

This is room four of the ancient DNA laboratory. In this room, we prepare our extracted DNA for sequencing. In order to do that, there are multiple steps.

*{Text appears on screen: “Together, these steps are known as DNA Library Preparation.”}*

The first step we’re going to do right now, which is called a partial UDG treatment. What this basically means is that our DNA is very degraded. We want to remove some of that degradation. The ends of our DNA sequencing reads have a lot of damage. This treatment will basically remove some of the ends of our DNA reads to make them more readable later on for data analysis. Our first step here is to always UV all of the tubes that we’re going to be using for our analysis. We’re going to move them here into our UV crosslinker. *{Beeps from UV Crosslinker}* Now that our tubes have been UV, they’re now safe to work with. We’re now going to begin our partial UDG treatment. This process requires the use of specific reagents that are shown here.

*{Video pauses while the researcher is showing the reagent tubes, and the following caption is shown: “These reagents include an enzyme that helps attach the adapters onto the DNA fragments (T4 DNA ligase), buffers (including PEG-4000), and water.”}*

What we’re going to do is, we’ve already pre-made what is called a master mix, or basically a collection of all those reagents into one tube. What we’re going to do now is pipette out this master mix into each of the samples. This is called a PCR strip tube. It’s much, much smaller than our other tubes, and it allows us to put these little tubes into a special machine. We have to use these small types of tubes. Now we’ve been able to add our master mix. Now we’re going to take our DNA samples. I’m going to add about 30 microliters. And we’re also going to use a water blank to make sure that our process is clean. This acts as a negative control. So now we have three samples in total, and we’re going to take these DNA samples over to incubate.

Video 2 Removing Ancient DNA Damage – Incubation

Now we’re going to take our partial UDG libraries, this is our DNA extract from our first step, place it into this machine called a thermocycler, put the lid down, and we’re going to set a specific temperature. I’m going to say “Run,” then “Okay,” and now this is going to take it through an incubation for 30 minutes at 37 degrees Celsius followed by a cool down.

So now our incubation is finished. What has happened to the DNA is that the enzymes present in our sample have been able to eat and chew away at the ends of our DNA fragments which is exactly what we want so we can make them easier to read for sequencing.

Video 3 Removing Ancient DNA Damage – UGI Treatment

Now we’re going to take our incubated DNA that’s all been chewed up as part of our first step and we’re going to now inactivate that enzyme. What we’re going to do is use something called a UGI inhibitor, which is going to stop the enzyme from working. So, what we’re going to do is take this inhibitor and add it to our DNA sample.

So, we’ve now put this DNA through another incubation step. This incubation now functions to stop that enzyme from chewing that DNA. This is what we call an inactivation step. It stops the enzymes from working and this is the end of our first step of library preparation.

Video 4 End Repair and Adapter Ligation

So, we’re now going to be in our second step of library preparation, where we make our DNAs ready to be read on a sequencing machine. The next step to do what is called an end repair. What this means is that, you know, we’ve damaged the ends of our DNA bases. Now we need to repair them and make them nice and whole and clean. So, we’re going to do that by again using a series of reagents.

*{Video pauses while the researcher is showing the reagent tubes, and the following caption is shown: “These reagents include two enzymes that repair the ends of the DNA fragments (T4 DNA polymerase and T4 Polynucleotide Kinase), individual DNA nucleotides (dNTPs), buffer, and water.”}*

So, what we’re going to do is take these reagents, and make them again into one master mix, which is combined small volumes of all those into this tube. And we’re going to pipette a certain amount of this into our DNA samples.

We’ve now added the master mix to our DNA samples, and we’re going to take them over again to our incubation machine and set them to incubate.

We finished our incubation step. Now our DNA is repaired. Now what we can do is add on what are called adapters. These are small sequences of DNA. Think of them like tags. We have to tag our DNA with these specific sequences, which allow it to be read by the sequencing machine. So now our DNA is all prepped and ready to go, and we can add those special DNA tags to the end of our DNA fragments.

Alright, so the first step to add these DNA tags is to get the tags themselves. So, these are our DNA tags. This may just look like two small little tubes, but what they do is they contain what are called the adapters. So, what we’re going to do is pipette these adapters into our DNA samples. Alright, now our adapters are added to our DNA. What we’re going to do now is add a lot of some other reagents here, which help us attach those adapters to the DNA.

*{Video pauses while the researcher is showing the reagent tubes, and the following caption is shown: “These reagents include individual DNA nucleotides (dNTPs), ATP, water, a buffer (to control pH levels), and the enzyme (USER) that eats the damaged parts of the DNA fragments.”}*

So again, we’re going to make that into a pre-made master mix, which we’re going to aliquot into these tubes.

So now are all the reagents that we need for this reaction to happen, where we put the adapters onto our DNA, is ready to go. And we’re going to walk this now for another incubation step.

We’ve now finished our adapter ligation. So now our adapters are nice and fit and snug onto our DNA samples.

Video 5 PCR Preparation

So now we’re beginning the last step of library preparation. In this step, what we do is we basically try to take our DNA and make thousands and thousands of copies of this DNA. And that’s because what’s in these tubes is usually in very low concentration. The DNA that we get from an archaeological individual is usually very damaged and very low concentration DNA. So now what we have to do is just make a lot of it. So, we’re going to do something called PCR or polymerase chain reaction. And what this does is basically takes our DNA and makes thousands of copies of it through the uses of enzymes and special heat and cycles.

So before we do that though, what we’re going to do is we’re going to take this DNA and we’re going to do a wash. This is exactly like what we did during DNA extraction. We’re going to take this solution and clear off whatever else got stuck in this DNA, including extra reagents, extra adapters, things that we just don’t need for our PCR reaction.

So now our DNA has been washed. We have them now in these larger tubes and we have clean DNA that’s ready for amplification. Now also importantly, in this step we’re going to tag each DNA sample with two special and unique indexing adapters. These two act like a naming. We’re going to name this DNA using these two special DNA tags. Every sample will get its own tag. So, this will go here, this one will get these tags, and this one will get these tags. And this will help us differentiate between all of our samples, so we know that they’re different from each other. So now what we’re going to do is first add our DNA tags into our small PCR tubes. What this is going to do is just make sure for each pair we have the right tags. Now what we’re going to do is we’re going to do many replicates of the same kind. We have four different replicates. This allows us to maximize our PCR yield. So, every one of these is the same sample. We just have them four times. Now what we’re going to do is do our next DNA tag for that same sample.

Alright, now we’re ready to add our master mix. This master mix is a combination of these reagents, which includes a DNA polymerase and a serum to help that polymerase and the reaction run smoother. So, we’ve already made this master mix. It’s present in here, and what we’re going to do is aliquot this into each one of our samples. Okay, we’re now going to repeat that process for all the rest of these samples. Now what we’re going to do is add in our DNA samples, our cleaned filtered DNA samples with our little adapters that are already on there. We’re going to add them to each of these small tubes, and we’re going to repeat the process for each one of these samples. So now our process is complete. We have our DNA samples loaded into each one of these tubes. So, we can now put this and we’re going to take them into the modern lab to do the polymerase chain reaction to amplify this DNA.

**View 8 Sequencing Center at UConn**

***View 8 Description:*** The final view shows UConn’s Center for Genome Innovation sequencing center, where DNA libraries are sequenced. Orientation Audio introduces the facility and its role in sequencing data generation. The space includes Illumina sequencing instruments and computers, with two researchers (Fleskes and Bolnick) standing at a large white Illumina NovaSeq 6000 sequencing machine. One Information Hotspot describes the Illumina MiSeq sequencing machine and its function in imaging nucleotide bases to generate DNA sequencing data. An Embedded Skills Video explains the DNA sequencing process and the types of research questions that can be addressed through aDNA analyses. Users may return to the beginning of the tour by clicking the white navigation arrow labeled, ‘Return to Beach Hall.’

***View 8 Orientation Audio:*** *“This the University of Connecticut’s Center for Genome Innovation. Here at this sequencing center, our DNA libraries are read by a sequencing machine in order to obtain DNA sequence information.”*

***View 8 Information Hotspots:***

| Information Hotspots | Accompanying Descriptive Text |
| --- | --- |
| 1: Illumina MiSeq Sequencing Machine | *“This is an Illumina MiSeq Sequencing Machine, which images the bases (A, G, C, T) in our DNA to generate sequencing data”* |

***View 8 Embedded Skills Videos Transcripts:***

Video 1 From aDNA Sequence to Beyond

Now, once the DNA libraries are ready, the real magic begins. Sequencing. This is the moment when we turn invisible molecules into data that we can actually see, measure, and interpret.

It’s a bridge between the biological and the digital worlds, where chemistry and computation work hand in hand.

And we use what’s called next generation sequencing, or NGS for short. The next generation part refers to how this technology reads millions of DNA fragments at the same time, earlier methods could only sequence one piece of DNA at a time, like reading a book letter by letter. Next generation sequencing is more like opening a library and scanning a million pages all in parallel, which is pretty amazing. This shift has completely transformed genetics. It’s made sequencing faster, cheaper, and far more powerful, opening doors from everything from personalized medicine to ancient DNA research, which is the subject of this video.

Now, sequencing takes place on a small glass slide called a flow cell. On its surface, a thin chemical layer, sometimes called a “lawn,” covered with tiny little anchors made of short DNA strands. Each of these anchors is waiting for one DNA fragment from our sample to land on it and stick it, like Velcro almost for molecules. When a fragment attaches, it bends and copies itself many, many, many times over in order to create a tiny cluster of identical DNA strands, all derived from one original molecule. Millions of these clusters cover the entire flow cell, and each one will be read individually. Once the flow cell is prepared, the machine adds a special chemical version of the DNA building blocks, the A’s, T’s, C’s, and G’s, each tacked with a different fluorescent color. So, every time one of these bases is added to a growing strand, it gives off a quick flash of light. High-speed cameras inside the sequencer capture those flashes in real time, taking thousands of pictures per second. The pattern of colors becomes the code that tells us which bases were added and in what order.

This process repeats for every cluster on the flow cell, layer by layer, until the instrument has read millions of fragments base by base. All those flashes of light are then converted into digital signals through the sequencer software, turning those images into strings of A’s, T’s, G’s, and C’s, right? The digital version of our DNA sequence. Now those strings of data are stored in something called a FASTQ file, which holds not only just the sequences, but also the information about how confident the machine was in reading each base that was called.

These files are massive, sometimes hundreds of gigabytes, maybe even terabytes, and represent millions or even billions of individual reads. In this way, sequencing is both a scientific and a storytelling process. It translates the chemistry of life into digital forms, allowing us to explore histories that are microscopic, molecular, but also really deeply human.

Every sequence begins as a tiny fragment of DNA, but once it’s digitized, it becomes something larger. Part of a vast record of life’s diversity, past and present. Sequencing lets us read those stories one molecule at a time, millions of times over.

When the sequencer finishes its run, we’re left with millions, sometimes billions, of short DNA reads. Each one is a fragment, just a few dozen or a hundred base pairs long. On their own, they don’t say much, but together they hold untold knowledge of ancient lives.

But sequencing and analysis are only one part of the story. The real power of this work lies in how we interpret these molecular histories, what they tell us about identity, movement, and connection across generations. To explore that side more, we’ll turn now to Dr. Bolnick, who will reshare how these genetic and microbial stories deepen our understanding of the past, and reshape questions that we ask as anthropologists.

Once we reconstruct the sequence of letters for one person, we can then line up and compare DNA sequences for multiple individuals. For example, here we can see partial DNA sequences for five individuals. We can see that Individuals 1 and 2 exhibit the same series of letters for this strand of DNA, whereas Individual 3 differs from the first two at two positions, position two and position nine here. Points where there is a single letter difference in the DNA sequence are called single nucleotide polymorphisms, or SNPs, which is really just a fancy way of saying that there’s a variation in which nucleotide or which letter is present at that point in the DNA from person to person.

*{Text appears on screen: “Single nucleotide polymorphisms, or SNPs, are a genetic variation of a difference in only one DNA building block (i.e., nucleotide).”*

When two people exhibit exactly the same DNA sequence in a section of DNA that we’re interested in, we say that they share a haplotype, or a specific DNA sequence. If we look at the sequences for these five individuals here, we see that Individuals 1 and 2 exhibit the same DNA sequence. They share a haplotype. Individuals 3 and 5 share a different haplotype.

Sometimes scientists also use the term haplogroup to refer to a group of similar but slightly different DNA sequences. For example, if we take the sequences for these five individuals, we might say that Individuals 1, 2, 3, and 5 belong to the same haplogroup because they all exhibit very similar DNA sequences, whereas Individual 4 has a more different sequence and thus belongs to a different haplogroup.

These similarities and differences in the DNA can help shed light on the degree of relatedness between two individuals or groups of people, as well as on their ancestry. The process of inferring ancestry and relatedness for individuals who lived in the past is quite similar to what ancestry testing companies like 23andMe or Ancestry.com do for living individuals today. To determine relatedness, we look at how similar or how different the DNA is for two or more people. If two people share long stretches of identical or very similar DNA, they are likely closely related. If they have less DNA in common, they’re only more distantly related to one another.

The process of inferring ancestry, though, for ancient individuals is a little bit more complicated.

Ideally, to directly assess an ancient person’s ancestry, we would need DNA from people who lived earlier in time than our research subjects and in the locations where their ancestors might have come from. However, because there is not a lot of ancient DNA available for many, many people around the world yet, we rarely have that kind of detailed information. So instead, what often happens now is that we will look at DNA from an ancient individual and compare their DNA to DNA from people alive today who live in various locations around the world, and then identify closely related individuals in that database. In other words, we’re looking for people in this database who share large sections of DNA with the ancient individual that we’re studying, which they presumably inherited from a shared ancestor or ancestors. If we assume that the shared ancestors lived in the same location as those alive today, we can then infer that our research subjects, the ancient individuals, likely have ancestry from that particular region or particular community.

It’s worth noting, though, that those ancestors may not have held the same social identity or had the same ethnic or cultural or national affiliation as their descendants. To really understand how ancient ancestors might have identified, we also need other kinds of information coming from historical documents, archaeological context, genealogical information, to help us evaluate those kinds of affiliations.

When we sequence DNA from an ancient individual, we’re unlikely to sequence that person’s entire genome in completion. Because DNA begins to degrade and break down as soon as a person dies, the DNA that’s found in an ancient individual’s remains is broken up, it’s fragmented, it may have been damaged and changed chemically after that person’s death. So, the DNA that we retrieve from a DNA sequencer will be fragmented, it will be incomplete, and we’re therefore going to work with only the fragments that we can retrieve, and that’s what we’re limited to for comparisons with other individuals.

One of the common types of DNA that we can look at in ancient individuals is mitochondrial DNA. This is DNA that we inherit only from our mothers, and there are many copies of mitochondrial DNA in each cell in the body, so it’s more likely to survive after an individual dies in their remains.

Mitochondrial DNA is maternally inherited, so if we look at the mitochondrial DNA sequence for two individuals and find that they exhibit the same sequence or the same haplotype, that can indicate that they share a direct maternal ancestor. What we don’t know from that sharing though is exactly how far in the past that shared ancestor lived. They might exhibit the same mitochondrial sequence, the same haplotype, because they have the same mother. In contrast, they might exhibit the same mitochondrial haplotype because their mother’s mother’s mother’s mother was in common, or the shared ancestor could have lived even farther back in the past.

Ideally, we would want to look at more than just the mitochondrial DNA, to look at DNA inherited from both parents to get a bigger picture of relatedness and a better assessment of how closely related two individuals are.

When we compare the DNA sequences, the haplotypes and the haplogroups among individuals, we can look to see where in the world these sequences are most common. There is geographic patterning to this where some haplotypes are more common in some parts of the world than others, and by looking to see where a haplotype is present, where it’s common around the world, that can give us some insight into where a person’s ancestors may have come from.
